# Towards the Development of a Conceptual Framework of the Determinants of Pre‐Eclampsia: A Hierarchical Systematic Review of Biomarkers

**DOI:** 10.1111/1471-0528.18194

**Published:** 2025-05-08

**Authors:** Terteel Elawad, Hiten D. Mistry, Mai‐Lei Woo Kinshella, Marianne Vidler, Marie‐Laure Volvert, Helen Elwell, Veronique Filippi, Kelly Pickerill, Rachel Craik, Joel Singer, Rosemary Townsend, Hannah Blencowe, Eleni Z. Tsigas, Jeffrey N. Bone, Peter von Dadelszen, Laura A. Magee, the PRECISE Network, Umberto D’Alessandro, Anna Roca, Hawanatu Jah, Andrew Prentice, Melisa Martinez‐Alvarez, Brahima Diallo, Abdul Sesay, Sambou Suso, Baboucarr Njie, Fatima Touray, Yahaya Idris, Fatoumata Kongira, Modou F. S. Ndure, Lawrence Gibba, Abdoulie Bah, Yorro Bah, Marleen Temmerman, Angela Koech, Patricia Okiro, Geoffrey Omuse, Grace Mwashigadi, Mary Goretti Amondi, Consolata Juma, Joseph Mutunga, Moses Mukhanya, Robin Okello, Onesmus Wanje, Isaac Mwaniki, Marvin Ochieng, Emily Mwadime, Esperança Sevene, Corssino Tchavana, Salesio Macuacua, Anifa Vala, Helena Boene, Lazaro Quimice, Sonia Maculuve, Inacio Mandomando, Peter von Dadelszen, Laura A. Magee, Rachel Craik, Marie‐Laure Volvert, Hiten Mistry, Thomas Mendy, Donna Russell, Prestige Tatenda Makanga, Liberty Makacha, Reason Mlambo, Lucilla Poston, Rachel Tribe, Sophie Moore, Tatiana Salisbury, Aris Papageorghiou, Alison Noble, Rachel Craik, Hannah Blencowe, Veronique Filippi, Joy Lawn, Matt Silver, Joseph Akuze, Ursula Gazeley, Judith Cartwright, Guy Whitley, Sanjeev Krishna, Marianne Vidler, Jing Li, Jeff Bone, Mai‐Lei Woo Kinshella, Domena Tu, Ash Sandhu, Kelly Pickerill, Carla Carillho, Benjamin Barratt

**Affiliations:** ^1^ Department of Women and Children's Health School of Life Course Sciences, Faculty of Life Sciences and Medicine, King's College London London UK; ^2^ Department of Population Health Sciences College of Life Sciences, University of Leicester Leicester UK; ^3^ Department of Obstetrics and Gynaecology BC Children's and Women's Hospital and University of British Columbia Vancouver British Columbia Canada; ^4^ BMA Library British Medical Association London UK; ^5^ Faculty of Epidemiology and Population Health, London School of Hygiene and Tropical Medicine London UK; ^6^ School of Population and Public Health, Centre for Health Evaluation and Outcome Sciences University of British Columbia Vancouver British Columbia Canada; ^7^ Centre for Reproductive Health Institute for Regeneration and Repair, University of Edinburgh Edinburgh UK; ^8^ Preeclampsia Foundation Melbourne Florida USA

**Keywords:** Biomarkers, Conceptual framework, Pre‐eclampsia

## Abstract

**Background:**

Pre‐eclampsia is a leading cause of maternal and perinatal morbidity and mortality. There are several determinants of individual pregnant women's risk of developing pre‐eclampsia, including biomarkers and ultrasound markers.

**Objective:**

A conceptual framework to collate and summarise the extensive body of literature on biomarkers (including ultrasound markers) associated with pre‐eclampsia, through a hierarchical systematic literature review.

**Search Strategy:**

Medline, Embase, Health Technology Assessments, Database of Abstracts of Reviews of Effects, Cochrane Library were searched until April 2024.

**Selection Criteria:**

Reviews and cohort studies (> 100 participants) reporting biomarkers associated with pre‐eclampsia were included.

**Data Collection and Analysis:**

Studies were screened by title, then abstract and full text. Evidence was prioritised from umbrella reviews, followed by systematic reviews and then observational studies. Associations were assessed for strength of association and quality of evidence using GRADE.

**Main Results:**

The biomarker domain included 40 individual determinants of pre‐eclampsia. Of these, there were 18 biomarkers with definite or probable associations based on moderate‐strong quality evidence across markers of angiogenic imbalance, fetal‐placental unit function, inflammatory and immune markers, and physiological markers. Vascular endothelial growth factor, human chorionic gonadotropin, inhibin‐A, maternal serum placental protein‐13, and interferon‐gamma had definite associations based on high‐quality evidence.

**Conclusion:**

Biomarkers associated with the development of pre‐eclampsia highlight the multi‐factorial aetiology of the syndrome. The addition of biomarkers, including ultrasound, will optimise the prediction of pre‐eclampsia and enable individualised risk stratification.

## 1 Introduction

Pre‐eclampsia is a severe pregnancy complication, distinguished by the emergence of *de novo* hypertension after 20 weeks of gestation, accompanied by proteinuria and/or evidence of maternal acute kidney injury, liver dysfunction, neurological features, haemolysis or thrombocytopaenia or fetal growth restriction [1]. Globally, it ranks as the second most prevalent cause of maternal mortality, resulting in more than 46 000 maternal and 500 000 perinatal deaths annually [2]. This burden is particularly pronounced in low‐ and middle‐income countries. Furthermore, pre‐eclampsia is associated with lifelong consequences for both the mother and her child, including increased risks of cardiovascular, renal and metabolic disease [2, 3].

As the pathophysiology of pre‐eclampsia remains to be fully elucidated, there is a need to further understand the determinants to develop and improve risk screening and preventative strategies [4]. Building on extensive research and the development of several biomarkers for pre‐eclampsia that reflect the heterogeneous nature of the syndrome [4, 5], cost‐effective, safe, and reliable methods to predict pre‐eclampsia have been developed and, for preterm disease, have guided effective evidence‐based interventions [6, 7, 8, 9].

The aim of this study was to develop a conceptual framework that systematically summarises the current high‐quality evidence in relation to biomarkers, including ultrasound markers, of pre‐eclampsia risk. A conceptual framework maps the literature on biomarkers predictive of the development of pre‐eclampsia by the strength of association and quality of the evidence and has the potential to inform prevention strategies and risk stratification to guide surveillance and care pathways.

## 2 Material and Methods

Detailed methodology for this study has been previously described [10], but is described briefly below. The biomarkers conceptual framework is part of the larger PRECISE conceptual framework on determinants of pre‐eclampsia.

### 2.1 Search Strategy

We employed the methods of Hiatt et al. [11], to develop a criteria‐based model of determinants using a systematic process with the aim of building a conceptual framework to describe a comprehensive multi‐factorial model of biomarkers (including ultrasound markers), determinants of pre‐eclampsia. A broad working model of known determinants was assembled by the ‘PREgnancy Care Integrating translational Science, Everywhere’ (PRECISE) Network [12] (Table S1) based on variables found to have significant associations with pre‐eclampsia from pooled results from umbrella reviews of systematic reviews [13, 14]. The search strategy was developed in consultation with a clinical librarian at the British Medical Association (HE), and designed to identify the highest level of evidence. Detailed nutritional biomarkers have previously been published by our group [15] and thus not included in this search. Systematic searches were conducted on Medline, Embase, Evidence‐Based Medicine Reviews (Health Technology Assessments, Database of Abstracts of Reviews of Effects, Cochrane Library databases), Google Scholar, and reference lists from the database inception to April 2024 for relationships between biomarkers and pre‐eclampsia. Medical subject heading and free text words were used to extract relevant studies from the database; search terms are reported in Table S2.

Following the methods of Hiatt et al. [11], studies were selected according to a hierarchy of evidence that prioritised umbrella reviews (systematic reviews of reviews), followed by systematic reviews with meta‐analyses and finally, large (at least 100 participants), observational cohort studies. Cohort studies with fewer than 100 participants, cross‐sectional surveys, case‐controlled studies, case reports/series, qualitative reviews, and editorials were excluded.

### 2.2 Data Extraction

Titles and abstracts of articles were screened by the review team (TE, MWK, HDM) to assess eligibility, with all potentially eligible studies undergoing full‐text review. Studies were included if they reported on biomarkers associated with the incidence of pre‐eclampsia. Studies that reported only on other forms of hypertensive disorders of pregnancy or pregnancy hypertension in general were excluded. Data were abstracted from umbrella reviews and their source reviews where applicable, individual reviews and cohort studies. Abstracted data included general study characteristics and strength of association between each biomarker and pre‐eclampsia and were expressed as odds ratios (OR), relative risk (RR), as reported in the reviews or individual studies. In addition, diagnostic OR (DOR), likelihood ratios (LR) and area under the receiver operating characteristic (AUROC) curve were used when included.

### 2.3 Quality of the Evidence

The Grading of Recommendations, Assessment, Development, and Evaluation (GRADE) [16, 17] approach was used to assess the quality of evidence, using four levels: high, moderate, low, and very low. Umbrella or systematic reviews are classed as high quality, compared to single observational studies which were considered low certainty of evidence that could be upgraded for large effect sizes or evidence of a dose– response relationship [17]. The certainty of the evidence was also lowered due to various factors including potential bias, inconsistency (significant variability *I* ^2^ > 50%), imprecise measurements (wide confidence intervals), and potential publication bias (asymmetric funnel plot).

The criteria for strength of association between each biomarker and pre‐eclampsia were based on point estimates of summary measures adapted from Hiatt et al. [11] (see Table 1). The strength of the relationship was categorised as definite (≥ 3.00 or < 0.33), probable (1.50–2.99 or 0.33–0.67), possible (1.10–1.49 or 0.68–0.89), and unlikely (0.90–1.09) We employed both RR and OR interchangeably for the model, as ORs provide a reasonable estimate of the RR when the outcome is observed in < 10% of both exposed and unexposed populations [18].

**TABLE 1:** Strength of association between risk factors and pre‐eclampsia based on point estimates of various summary measures.

	RR or OR^a^		DOR^b^	LR^c^		AUC point estimate^d^
	Decreases risk	Increases risk		LR+	LR−	
Definite	< 0.33	≥ 3.00	≥ 100	> 10	< 0.1	> 0.8
Probable	0.33–0.67	1.50–2.99	> 25 to < 100	5.01–10.0	0.10–0.19	0.70–0.80
Possible	> 0.67 to < 0.9	1.10–1.49	> 4 to ≤ 25	2.01–5.0	0.20–0.50	0.51–0.69
Unlikely	0.90–1.09		1–4	1.0–2.0	0.51–0.99	≤ 0.50

Abbreviations: LR, likelihood ratio; LR−, negative LR; LR+, positive LR; OR, odds ratio; RR, relative risk.

^a^ Based on Hiatt and modification of Harvard Cancer Risk Index.

^b^ Based on LR+ and LR− criteria and definition of DOR as LR+/LR−.

^c^ Based on UpToDate.

^d^ Based on Mandrekar J Thorac Oncol 2010. In general, an AUC of 0.5 suggests no discrimination (i.e., ability to diagnose patients with and without the disease or condition based on the test), 0.7 to 0.8 is considered acceptable, 0.8 to 0.9 is considered excellent, and more than 0.9 is considered outstanding.

The strength of association between variables based on DOR was categorised as follows following expert guidance from LAM/JS and based on Mahutte & Dulebi, 2024 [19]: definite: ≥ 100, probable: > 25 to < 100, possible: > 4 to ≤ 25, or not significant: 1–4. The strength of association between variables based on likelihood ratios (LR) was categorised according to UpToDate [19]. The following four categories were used: definite: > 10 or < 0.1, for a positive or negative likelihood ratio, respectively; probable: 5.01–10.0 or 0.10–0.19, for a positive or negative likelihood ratio, respectively; possible: 2.01–5.0 or 0.20–0.50, for a positive or negative likelihood ratio, respectively; or not significant: 1.0–2.0 or 0.51–0.99, for a positive or negative likelihood ratio, respectively.

The strength of association between variables based on AUROC curve, as reported in reviews or individual studies, was categorised according to diagnostic test assessment. In general, an AUROC of 0.5 suggests no discrimination (i.e., ability to diagnose patients with and without the disease or condition based on the test), 0.7 to 0.8 is considered acceptable, 0.8 to 0.9 is considered excellent, and more than 0.9 is considered outstanding [20]. The following four categories were used: definite: > 0.8, probable: 0.70–0.80, possible: 0.51–0.69, or unlikely: ≤ 0.50.

## 3 Results

Forty biomarkers were identified. Table 2 presents the framework of biomarkers according to their strength of association and quality of the evidence, with details on timing of measurement and onset of pre‐eclampsia where available (Data S1). Thirty‐eight biomarkers were based on evidence from umbrella reviews or systematic reviews [14, 21, 22, 23, 25, 26, 27, 28, 29, 30, 31, 32, 33, 34, 36, 37, 38, 39, 40, 41, 42]. Only two biomarkers were primarily based on evidence from a cohort study [35]. GRADE assessments for each biomarker are reported in Data S2.

**TABLE 2:** Summary of biomarkers for pre‐eclampsia, overall and by timing of measurement and pre‐eclampsia onset.

Biomarker	Effect estimate (95% CI)	*N* studies	*N* participants	*I* ^2^	Direction of effect	Strength of association	Certainty of evidence
Angiogenic imbalance							
PlGF [14, 21]	OR 0.36 (0.25, 0.54)	26	4425	84%	Higher levels as a protective factor	Probable	Moderate
Early onset PE^a^ [22]	OR 3.4 (1.6, 7.2)	4	1729	87%	Abnormal^b^ levels as a risk factor	Definite	Moderate
Late‐onset PE^a^ [22]	OR 1.85 (0.7, 4.33)	3	1262	89%	N/A	Unlikely	Low
sENG [14, 21]	OR 2.66 (1.53, 4.63)	19	3141	91%	Higher levels as a risk factor	Probable	High
Early onset PE^a^ [22]	OR 18.5 (8.4, 41.0)	2	2143	14%	Abnormal^c^ levels as a risk factor	Definite	High
Late‐onset PE^a^ [22]	OR 2.1 (1.9, 2.4)	3	2326	0%	Abnormal^c^ levels as a risk factor	Probable	High
sFlt‐1 [14, 21]	OR 2.38 (1.47, 3.86)	32	5230	93%	Higher levels as a risk factor	Probable	High
Early onset PE^a^ [22]	OR 1.2 (0.33, 4.41)	3	569	79%	N/A	Unlikely	Low
Late‐onset PE^a^ [22]	OR 1.03 (0.62, 1.74)	3	778	50%	N/A	Unlikely	Low
VEGF [14, 21]	OR 0.10 (0.01, 1.53)	4	265	96%	Higher levels as a protective factor	Definite	High
Markers of fetal placental unit function							
AFP ≥ 2.00 MoM (2nd trimester) [13, 23]	LR+ 2.36 (1.46–3.83) LR− 0.96 (0.95–0.98)	10	103 627	N/A	Higher levels as a risk factor	Possible	Low
AFP ≥ 2.00 MoM on preterm PE^d^ [24]	RR 2.6 (0.6, 11.1)	1	7929	N/A	N/A	Unlikely	Very low
AFP ≥ 2.00 MoM on term PE^d^ [24]	RR 1.1 (0.3, 4.5)	1	7929	N/A	N/A	Unlikely	Very low
β‐hCG [14, 25]	OR 88.7 (4.31, 1824)	12	8935	100%	Higher levels as a risk factor	Definite	High
1st‐trimester measurement [26]	SMD 0.002 (−0.12, 0.13)	15	24 591	76%	N/A	N/A	Low
2nd‐trimester measurement [26]	SMD 0.37 (0.19, 0.54)	6	3547	60%	Higher levels in PE group	N/A	Moderate
hCG > 2.0 MoM on preterm PE^d^ [24]	RR 3.8 (1.5, 9.8)	1	7929	N/A	Higher levels as a risk factor	Definite	Moderate
hCG > 2.0 MoM on term PE^d^ [24]	RR 2.0 (0.9, 4.2)	1	7929	N/A	N/A	Unlikely	Very low
Inhibin‐A (1st trimester) [14, 22]	OR 3.57 (1.68, 7.61)	3	1215	21%	Higher levels as a risk factor	Definite	High
Early onset PE^a^ [22]	OR 4.1 (1.9, 8.8)	1	234	N/A	Higher levels as a risk factor	Definite	Moderate
Late‐onset PE^a^ [22]	OR 1.9 (1.4, 2.8)	2	441	0%	Higher levels as a risk factor	Probable	Low
PAPP‐A (1st trimester) [14, 22]	OR 2.05 (1.62, 2.59)	9	53 355	45%	Low levels as a risk factor	Probable	High
Early onset PE^a^ [22]	OR 4.8 (2.5, 22.5)	4	9713	72%	Low levels as a risk factor	Definite	Moderate
Late‐onset PE^a^ [22]	OR 1.44 (0.89, 2.32)	2	8726	0%	N/A	Unlikely	Low
PP13 (1st trimester) [14, 22]	OR 4.43 (2.86, 6.85)	4	4061	49%	Low levels as a risk factor	Definite	High
Early onset PE^a^ [22]	OR 7.5 (2.5, 22.5)	3	3184	69%	Low levels as a risk factor	Definite	High
Markers of lipid metabolism and oxidative stress							
HDL‐c (1st & 2nd trimester, mg/dL) [13, 27]	WMD −0.48 (−3.31, 2.34)	10	7369^e^	73%	N/A	N/A	Low
HDL‐c (3rd trimester, mg/dL) [13, 27]	WMD −8.86 (−11.50, −6.21)	41	7369^e^	95%	Lower levels in PE group	N/A	Low
LDL‐c (1st & 2nd trimester, mg/dL) [13, 27]	WMD 3.89 (−0.19, 7.97)	9	7369^e^	13%	N/A	N/A	Moderate
LDL‐c (3rd trimester, mg/dL) [13, 27]	WMD 10.92 (−0.59, 22.42)	36	7369^e^	97%	N/A	N/A	Low
Total cholesterol (1st & 2nd trimester, mg/dL) [13, 27]	WMD 12.49 (3.44, 21.54)	11	7369^e^	56%	Higher levels in PE group	N/A	Low
Total cholesterol (3rd trimester, mg/dL) [13, 27]	WMD 20.20 (8.70, 31.70)	46	7369^e^	99%	Higher levels in PE group	N/A	Low
Triglycerides (1st & 2nd trimester, mg/dL) [13, 27]	WMD 25.08 (14.39, 35.77)	13	7369^e^	90%	Higher levels in PE group	N/A	Low
Triglycerides (3rd trimester, mg/dL) [13, 27]	WMD 80.29 (51.45, 109.13)	44	7369^e^	94%	Higher levels in PE group	N/A	Low
Ischaemia‐modified albumin [28]	OR 3.38 (2.23, 4.53)	3	206	0%	Higher levels in PE group	Probable	High
Uric acid [28]	OR 3.05 (2.39, 3.71)	3	267	0%	Higher levels in PE group	Probable	High
Malondialdehyde [28]	OR 2.37 (1.03, 3.70)	3	224	16%	Higher levels in PE group	Probable	High
Inflammatory and immune markers							
CRP [29]	SMD 0.52 (0.34, 0.69)	6	885	24%	Higher levels in PE group	N/A	Moderate
IL‐4 [29]	SMD 0.25 (0.076,0.433)	2	437	0%	Higher levels in PE group	N/A	Moderate
IL‐6 [29]	SMD 0.60 (0.36, 0.83)	10	1236	70%	Higher levels in PE group	N/A	Moderate
IL‐8 [29]	SMD 0.53 (0.28, 0.77)	4	431	45%	Higher levels in PE group	N/A	Moderate
TNF‐α [29]	SMD 0.59 (0.34, 0.83)	9	1331	74%	Higher levels in PE group	N/A	Low
IL‐18 [14, 30]	OR 1.13 (0.49, 2.60)	10	772	89%	N/A	Unlikely	Low
IFN‐γ [14, 30]	OR 5.42 (1.14, 25.7)	12	1268	97%	Higher levels as a risk factor	Definite	High
HLA antibodies [14, 31]	OR 0.93 (0.09, 9.77)	3	337	66%	N/A	Unlikely	Very low
Anticardiolipin antibodies ≥ 20 units [14, 32]	OR 2.85 (1.37, 5.95)	12	6747	69%	Higher levels as a risk factor	Probable	Moderate
sHCA‐G (1st trimester) [33]	SMD −0.84 (−1.29, −0.38)	3	643	54%	Lower levels in PE group	N/A	Low
sHCA‐G (2nd trimester) [33]	SMD −0.48 (−1.16, 0.20)	3	346	75%	N/A	N/A	Very low
sHCA‐G (3rd trimester) [33]	SMD −0.39 (−0.71, −0.06)	7	950	79%	Lower levels in PE group	N/A	Very low
ABO blood group							
O blood group [34]	OR 0.95 (0.93, 0.97)	12	714 153	18%	N/A	Unlikely	High
AB blood group [34]	OR 1.46 (1.12, 1.91)	8	709 623	62%	Higher in PE group	Possible	Moderate
A blood group [34]	OR 1.02 (0.90, 1.16)	8	409 623	49%	N/A	Unlikely	Moderate
B blood group [34]	OR 1.02 (0.98, 1.05)	8	709 623	0%	N/A	Unlikely	Moderate
Physiological markers							
Early pregnancy^f^ sBP 120–129 and dBP < 80 mmHg [35]	RR 2.38 (1.88, 3.02)	1	1929	N/A	Lower in PE group	Probable	High
Early pregnancy^f^ sBP 130–139 mmHg or dBP 80–89 mmHg [35]	RR 2.94 (2.34, 3.71)	1	1871	N/A	Higher in PE group	Probable	High
Arterial stiffness [14, 36]	OR 18.6 (3.72, 93.0)	9	845	93%	Increased levels as a risk factor	Definite	Moderate
Serum concentration of NO (μmol/mL) [14, 37]	OR 0.17 (0.04, 0.81)	9	600	95%	Higher levels as a protective factor	Definite	Moderate
Uterine artery Doppler—FVW (1st trimester) [13, 38]	LR+ 4.0 (2.7, 6.0) LR− 0.79 (0.74, 0.84)	8	37 971	N/A	Abnormal FVW as a risk factor	Possible	Moderate
Early onset PE^a^ [38]	LR+ 6.1 (4.1, 8.9) LR− 0.57 (0.48, 0.67)	7	38 611	N/A	Abnormal FVW as a risk factor	Probable	Moderate
Late‐onset PE^a^ [38]	LR+ 2.2 (1.9, 2.6) LR− 0.87 (0.83, 0.91)	3	33 879	N/A	Abnormal FVW as a risk factor	Possible	Moderate
Ophthalmic artery Doppler—peak ratio [39]	AUC 0.89	6	866	N/A	Higher peak ratio as a risk factor	Definite	Low
Ophthalmic artery Doppler–second peak systolic velocity [39]	AUC 0.93	3	425	N/A	Higher second peak as a risk factor	Definite	Low
Other							
Female fetal sex [13, 40]	OR 1.04 (0.97, 1.12)	11	219 575	39%	N/A	Unlikely	Low
Early onset PE^a^ [40]	OR 1.36 (1.17, 1.5)	11	219 575	21%	Higher in PE group	Possible	Moderate
Preterm PE^d^ [40]	OR 1.17 (1.02, 1.35)	11	219 575	33%	Higher in PE group	Possible	Moderate
Term PE^d^ [40]	OR 0.96 (0.90,1.01)	11	219 575	0%	N/A	Unlikely	Low
Early pregnancy^f^ HbA1c > 39 nmol/mol [41]	RR 2.02 (1.53, 2.66)	5	28 203	0%	Higher levels as a risk factor	Probable	High
Mean platelet volume [42]	LR+ 2.58 (2.01, 3.31) LR− 0.38 (0.28, 0.52)	22	20 137	97%	Higher levels as a risk factor	Possible	Low

Abbreviations: CI, confidence interval; CRP, C‐reactive protein; DOR, diagnostic odds ratio; fl, femtoliters; FVW, flow velocity waveform; HbA1c, glycated haemoglobin; HLA—human leukocy, e antigen; *I* ^2^, measure of heterogeneity (*I* ^2^ > 50% significant variability between studies); IFN‐γ, interferon‐gamma; IL, interleukin; MOM, multiple of the median; NO, nitric oxide; OR, odds ratio; PAPP‐A, pregnancy‐associated plasma protein A; PC, platelet count; PE, pre‐eclampsia; PlGF, placental growth factor; PP13, placental protein 13; RR, relative risk; sENG, soluble endoglin; sFLT‐1, soluble fms‐like tyrosine kinase‐1; sHCA‐G, soluble human leukocyte antigen‐G; SMD, standard mean difference; TNF, tumour necrosis factor; VEGF, vascular endothelial growth factor; WMD, weighted mean difference; β‐hCG, beta human chorionic gonadotropin. Strength of association: increasing depth of green fill to cells reflects increasing strength of association. Certainty of evidence: increasing depth of tan reflects incerasing certainty of evidence.

^a^ Early onset pre‐eclampsia < 34 weeks; late‐onset pre‐eclampsia ≥ 34 weeks.

^b^ As reported in the source reference listed by biomarker; likely lower levels as a risk factor.

^c^ As reported in the source reference listed by the biomarker; likely higher levels as a risk factor.

^d^ Preterm pre‐eclampsia < 37 weeks; term pre‐eclampsia ≥ 37 weeks.

^e^ Total sample of the review; numbers not reported for separate biomarkers.

^f^ < 20 week.

Nine biomarkers were identified as having a definite association with pre‐eclampsia, including vascular endothelial growth factor (VEGF) [14, 21], beta human chorionic gonadotropin (β‐hCG) [14, 25], inhibin‐A [14, 22], placental protein 13 (PP13) [14, 22], and interferon‐gamma (IFN‐γ) [14, 30], based on high‐quality evidence. Arterial stiffness [14, 36] and serum concentration of nitric oxide (NO) [14, 37] were based on moderate‐quality evidence due to heterogeneity, risk of bias, and imprecision, and upgraded for very large effect sizes. Higher peak ratio [39] and second peak systolic velocity [39] on ophthalmic artery Doppler were based on low‐quality evidence due to heterogeneity and potential imprecision (< 1000 total participants included in the meta‐analysis).

Eleven biomarkers had a probable association with pre‐eclampsia. Probable associations based on high‐quality evidence included soluble endoglin (sENG) [14, 21], soluble fms‐like tyrosine kinase‐1 (sFlt‐1) [14, 21], pregnancy‐associated plasma protein A (PAPP‐A) [14, 22], systolic blood pressure (sBP) 120–129 and diastolic blood pressure (dBP) above 80 mmHg before 20 weeks gestation [35], sBP 130–139 mmHg or dBP 80–89 mmHg before 20 weeks gestation [35], early pregnancy glycated haemoglobin (HbA1c) [41], ischaemia‐modified albumin [28], uric acid [28], and malondialdehyde [28]. Placental growth factor (PlGF) [14, 21] and anticardiolipin antibodies [14, 32] were probable associations based on moderate‐quality evidence. Evidence was downgraded for heterogeneity and potential publication bias, and upgraded for large effect sizes for both PlGF and anticardiolipin antibodies. Early pregnancy (< 20 weeks) elevated blood pressure demonstrated evidence of a dose effect, with larger effect sizes found for higher blood pressure levels [35].

Four biomarkers had a possible association with pre‐eclampsia. These included AB blood group [34] and first‐trimester abnormal flow velocity waveform (FVW) on uterine artery Doppler [13, 38], based on moderate‐quality evidence, and alpha‐fetoprotein (AFP) [13, 23] and mean platelet volume (MPV) [42] based on low‐quality evidence. Evidence was downgraded due to concerns about heterogeneity across the four possible biomarkers, a lack of reporting on publication bias for AFP, and risk of bias for MPV.

Six biomarkers were identified as unlikely to have an association with pre‐eclampsia. These included O blood group [34] based on high‐quality evidence, A blood group [34] and B blood group [34], based on moderate‐quality evidence due to wide confidence intervals (imprecision). IL (interleukin)‐ 18 [14, 30], female fetal sex [13, 40], and human leukocyte antigen (HLA) antibodies [14, 31] were based on low‐ to very low‐quality evidence due to concerns about the risk of bias, heterogeneity, and/or wide confidence intervals.

Ten biomarkers' strength of association could not be determined due to statistics not based on point estimates of summary measures (i.e., OR, RR, LR and AUROC point estimates), as described in our methodology. These included HDL‐c [13, 27], LDL‐c [13, 27], total cholesterol [13, 27], triglycerides [13, 27], C‐reactive protein (CRP) [29], IL‐4 [29], IL‐6 [29], IL‐8 [29], tumour necrosis factor (TNF‐α) [29], and soluble human leukocyte antigen‐G (sHCA‐G) [33].

Comparisons between the timing of measurement and the onset of pre‐eclampsia were reported for a limited number of biomarkers. PlGF [22], hCG > 2.0 multiples of the median (MoM) [24], PAPP‐A [22], and female fetal sex [40] were significantly associated with early‐onset or preterm pre‐eclampsia, and unlikely to be associated with late‐onset or term pre‐eclampsia. First trimester uterine artery Doppler flow velocity waveforms had a probable association with early‐onset pre‐eclampsia, while only a possible association with late‐onset pre‐eclampsia [38]. Levels of sENG [22] and inhibin‐A [22] were associated with both early‐ and late‐onset pre‐eclampsia. There were no biomarkers identified specifically for late‐onset or term pre‐eclampsia. β‐hCG levels [26] may only be significantly associated with pre‐eclampsia when measured in the 2nd trimester (not the 1st), and women with pre‐eclampsia may have lower HDL‐c levels [13, 27] specifically in the 3rd trimester.

## 4 Discussion

Based on the systematic literature, this conceptual framework finds that the strongest biomarkers predictive of pre‐eclampsia are markers of angiogenic imbalance (PlGF, sENG, sFlt‐1, VEGF) and fetal placental unit function (β‐hCG, inhibin‐A, PAPP‐A, PP13), all with definite or probable associations based on moderate to high quality of evidence (Table 3). It is known that pre‐eclampsia is partly mediated by dysfunctional syncytiotrophoblast, placental dysfunction, and angiogenic imbalance [2, 43, 44].

**TABLE 3:** Summary of biomarkers for overall pre‐eclampsia by strength of association and quality of the evidence.

		Quality of the evidence			
		High	Moderate	Low	Very low
Strength of the association	Definite	Higher VEGF Higher β‐hCG Abnormal Inhibin‐A Abnormal PP13 Higher IFN‐γ	Arterial stiffness Higher serum concentration of NO	Higher peak ratio on ophthalmic artery Doppler Higher second peak systolic velocity on ophthalmic artery Doppler	
	Probable	Higher sENG Higher sFlt‐1 Abnormal PAPP‐A Early pregnancy sBP 120–129 and dBP < 80 mmHg Early pregnancy sBP 130–139 mmHg or dBP 80–89 mmHg Higher early pregnancy HbA1c Ischaemia‐modified albumin Uric acid Malondialdehyde	Higher PlGF Higher anticardiolipin antibodies		
	Possible		AB blood group Abnormal FVW on uterine artery Doppler	Higher AFP Higher MVP	
	Unlikely	O blood group	A blood group B blood group	IL‐18 Female fetal sex	HLA antibodies

*Note:* Green text associated with decreased risk; red text associated with increased risk.

Physiological markers, including elevated blood pressure in early pregnancy (sBP 120–129 and dBP < 80 mmHg; 130–139 mmHg or dBP 80–89 mmHg), arterial stiffness and serum NO, were also identified by our model with definite or probable associations based on moderate‐high quality of evidence. Several lipid metabolism and oxidative stress biomarkers and inflammatory and immune biomarkers demonstrated potential associations with the initiation of pre‐eclampsia, but the point estimates of various summary measures could not be applied to determine the strength of association in our methodology.

A combination of biomarkers, maternal history and risk factors has contributed to reliable and cost‐effective methods of prediction of pre‐eclampsia [6, 7, 8, 9]. For example, screening by maternal factors, uterine artery pulsatility index and serum PlGF predicted 90% of early‐onset pre‐eclampsia, 75% of preterm pre‐eclampsia and 41% of term pre‐eclampsia [6]. Responding to this risk with 150 mg aspirin nightly until 36 weeks' gestation cost‐effectively reduces the odds of preterm disease by 62% [7, 9]. Our standardised method of prioritising umbrella reviews over even very large cohort studies with randomised controlled trial support has resulted in downgrading the bodies of evidence that support the validated 1st and 3rd trimester screening competing risk models [6, 8]. An updated umbrella review that reflects these data is required as excellent quality evidence has been downgraded.

There are other limitations to this study. For a few studies, the strength of association could not be evaluated based on point estimates of various summary measures (OR, RR, DOR, LR and AUC point estimates) that had been determined in the methods. Instead, some were reported in terms of mean differences and sensitivity and specificity of prediction models. Future work should involve using a tool which can determine the strength of association for these studies and prevent the exclusion of promising studies.

The conceptual framework for the predictive determinants of pre‐eclampsia has several strengths. First, it allows the examination of the complex relationships that have not been undertaken previously for pre‐eclampsia. Second, the evidence used in the conceptual model is derived from published peer‐reviewed literature and can be updated with the latest evidence (e.g., competing risks models). Most of the evidence came from umbrella reviews or systematic reviews. Lastly, the conceptual framework highlights where evidence is lacking and requires further research.

## 5 Conclusion

In brief, this hierarchical systematic literature review integrated 40 biomarkers into a conceptual framework for pre‐eclampsia. These markers, with their known functions, provide additional potential for their use for stratifying pre‐eclampsia risk, as well as further insights into disease pathophysiology. These data provide the best summary evidence for biomarker choice that might guide the constituent components and selection of screening tests to guide ASA prescription, antenatal care pathways, or timing of birth. Our results highlight the biomarkers most strongly linked with pre‐eclampsia diagnosis, offering valuable guidance for future evidence‐based clinical investigations and interventions.

## Author Contributions

L.A.M., P.v.D., V.F., M.‐L.V., T.E., and H.D.M. conceptualised and designed the study. L.A.M., P.v.D., T.E., and H.D.M. developed the methodology, and T.E. conducted the data collection. T.E. and M.‐L.W.K. performed the data analysis and interpretation. T.E., M.‐L.W.K., and H.D.M. drafted the original manuscript. T.E., M.‐L.W.K., and H.D.M. reviewed and edited the manuscript for critical intellectual content. L.A.M. and P.v.D. supervised the research project. All authors read and approved the final version of the manuscript. The PRECISE Network contributed to study design and provided constructive challenges as part of the development of a suite of conceptual frameworks for placental disorders.

## The PRECISE Network

Collaborators: Umberto D’Alessandro, Anna Roca, Hawanatu Jah, Andrew Prentice, Melisa Martinez‐Alvarez, Brahima Diallo, Abdul Sesay, Sambou Suso, Baboucarr Njie, Fatima Touray, Yahaya Idris, Fatoumata Kongira, Modou F. S. Ndure, Lawrence Gibba, Abdoulie Bah, Yorro Bah, Marleen Temmerman, Angela Koech, Patricia Okiro, Geoffrey Omuse, Grace Mwashigadi, Mary Goretti Amondi, Consolata Juma, Joseph Mutunga, Moses Mukhanya, Robin Okello, Onesmus Wanje, Isaac Mwaniki, Marvin Ochieng, Emily Mwadime, Esperanca Sevene, Corssino Tchavana, Salesio Macuacua, Anifa Vala, Helena Boene, Lazaro Quimice, Sonia Maculuve, Inacio Mandomando, Peter von Dadelszen, Laura A. Magee, Rachel Craik, Marie‐Laure Volvert, Hiten Mistry, Thomas Mendy, Donna Russell, Prestige Tatenda Makanga, Liberty Makacha, Reason Mlambo, Lucilla Poston, Rachel Tribe, Sophie Moore, Tatiana Salisbury, Aris Papageorghiou, Alison Noble, Rachel Craik, Hannah Blencowe, Veronique Filippi, Joy Lawn, Matt Silver, Joseph Akuze, Ursula Gazeley, Judith Cartwright, Guy Whitley, Sanjeev Krishna, Marianne Vidler, Jing Li, Jeff Bone, Mai‐Lei Woo Kinshella, Domena Tu, Ash Sandhu, Kelly Pickerill, Carla Carillho, Benjamin Barratt.

## Ethics Statement

The review only utilised data from previously published studies.

## Conflicts of Interest

The authors declare no conflicts of interest.

## Supporting information


**Table S1** The PRECISE Network.
**Table S2** Search terms.

**Data S1** Summary of results.

**Data S2** GRADE assessments.

## Data Availability

The data that supports the findings of this study are available in the Supporting Information of this article.
