# Supplementary material for: Towards the Development of a Conceptual Framework of the Determinants of Pre‐Eclampsia: A Hierarchical Systematic Review of Biomarkers
Source: BJOG. 2025 May 8;133(Suppl 4):S30–42. doi: 10.1111/1471-0528.18194 (PMC13572888; doi:10.1111/1471-0528.18194)
Supplement: Supplementary file 1 [file BJO-133-S30-s003.docx]

**Supplementary Appendix**

|  | Title | Page number |
| --- | --- | --- |
| **Tables** |  |  |
| Table S1 | The PRECISE Network | 1 |
| Table S2 | Search terms | 2 |

**Table S1**: The PRECISE Network

| **In-country teams** | **Members** |
| --- | --- |
| THE GAMBIA: Medical Research Council Unit The Gambia at the London School of Hygiene and Tropical Medicine, Fajara | Umberto D’Alessandro, Anna Roca, Hawanatu Jah, Andrew Prentice, Melisa Martinez-Alvarez, Brahima Diallo, Abdul Sesay, Sambou Suso, Baboucarr Njie, Fatima Touray, Yahaya Idris, Fatoumata Kongira, Modou F.S. Ndure, Lawrence Gibba, Abdoulie Bah and Yorro Bah. |
| KENYA: Aga Khan University, Nairobi | Marleen Temmerman, Angela Koech, Patricia Okiro, Geoffrey Omuse, Grace Mwashigadi, Mary Goretti Amondi, Consolata Juma, Joseph Mutunga, Moses Mukhanya, Robin Okello, Onesmus Wanje, Isaac Mwaniki, Marvin Ochieng, Emily Mwadime |
| MOZAMBIQUE : Centro de Investigação em Saúde de Manhiça, Manhiça | Esperança Sevene, Corssino Tchavana, Salesio Macuacua, Anifa Vala, Helena Boene, Lazaro Quimice, Sonia Maculuve, Inacio Mandomando |
| **Central co-ordinating team** |  |
| Department of Women and Children’s Health, School of Life Course Sciences, Faculty of Life Sciences and Medicine, King’s College London | Peter von Dadelszen, Laura A. Magee, Rachel Craik, Marie-Laure Volvert, Hiten Mistry, Thomas Mendy |
| Donna Russell Consulting | Donna Russell |
| **Co-Investigator team** |  |
| Midlands State University, Zimbabwe | Prestige Tatenda Makanga, Liberty Makacha and Reason Mlambo |
| Kings College London | Lucilla Poston, Rachel Tribe, Sophie Moore, Tatiana Salisbury |
| University of Oxford | Aris Papageorghiou, Alison Noble, Rachel Craik |
| London School of Hygiene and Tropical Medicine | Hannah Blencowe, Veronique Filippi, Joy Lawn, Matt Silver, Joseph Akuze and Ursula Gazeley |
| St George’s, University of London | Judith Cartwright, Guy Whitley, Sanjeev Krishna |
| University of British Columbia | Marianne Vidler, Jing (Larry) Li, Jeff Bone, Mai-Lei (Maggie) Woo Kinshella, Domena Tu, Ash Sandhu, Kelly Pickerill |
| Eduardo Mondlane University, Maputo | Carla Carillho |
| Imperial College London | Benjamin Barratt |

**Table S2**: Details of search terms

exp Pre-Eclampsia/ exp Pre-Eclampsia/

(pre-eclampsia or preeclampsia).mp. [mp=title, abstract, original title, name of substance word, subject heading word, floating sub-heading word, keyword heading word, organism supplementary concept word, protocol supplementary concept word, rare disease supplementary concept word, unique identifier, synonyms]

1 or 2

exp Placenta Growth Factor/ or exp Pregnancy Proteins/

(placental growth factor or PlGF or PGF).mp. [mp=title, abstract, original title, name of substance word, subject heading word, floating sub-heading word, keyword heading word, organism supplementary concept word, protocol supplementary concept word, rare disease supplementary concept word, unique identifier, synonyms]

4 or 5

exp Pregnancy Proteins/ or exp Biomarkers/

(Soluble fms-like tyrosine kinase-1or sFlt-1 or sVEGFR-1).mp. [mp=title, abstract, original title, name of substance word, subject heading word, floating sub-heading word, keyword heading word, organism supplementary concept word, protocol supplementary concept word, rare disease supplementary concept word, unique identifier, synonyms]

7 or 8

exp Pregnancy Proteins/ or exp Pregnancy-Associated Plasma Protein-A/

(pregnancy*associated plasma protein A or Pappalysin-1 or PAPPA or PAPP-A).mp. [mp=title, abstract, original title, name of substance word, subject heading word, floating sub-heading word, keyword heading word, organism supplementary concept word, protocol supplementary concept word, rare disease supplementary concept word, unique identifier, synonyms]

10 or 11

exp Biomarkers/ or exp Pregnancy Proteins/

(Placental protein 13 or PP-13 or LGALS13).mp. [mp=title, abstract, original title, name of substance word, subject heading word, floating sub-heading word, keyword heading word, organism supplementary concept word, protocol supplementary concept word, rare disease supplementary concept word, unique identifier, synonyms]

13 or 14

(Vascular endothelial growth factor or VEGF or vascular permeability factor or VPF).mp. [mp=title, abstract, original title, name of substance word, subject heading word, floating sub-heading word, keyword heading word, organism supplementary concept word, protocol supplementary concept word, rare disease supplementary concept word, unique identifier, synonyms]

16 or 17

exp Endoglin/

(sEng or soluble endoglin).mp. [mp=title, abstract, original title, name of substance word, subject heading word, floating sub-heading word, keyword heading word, organism supplementary concept word, protocol supplementary concept word, rare disease supplementary concept word, unique identifier, synonyms]

19 or 20

exp Fetus/ or exp Sex Characteristics/ or exp Placenta/

female fetal sex.mp.

22 or 23

exp Ultrasonography, Doppler/

exp Uterine Artery/

(uterine artery doppler ultrasound or uterine artery doppler ultrasound or uterine artery doppler ultrasonography).mp. [mp=title, abstract, original title, name of substance word, subject heading word, floating sub-heading word, keyword heading word, organism supplementary concept word, protocol supplementary concept word, rare disease supplementary concept word, unique identifier, synonyms]

25 or 26 or 27

exp ABO Blood-Group System/

(blood group a or blood group b or blood group ab or blood group O or abo blood group).mp. [mp=title, abstract, original title, name of substance word, subject heading word, floating sub-heading word, keyword heading word, organism supplementary concept word, protocol supplementary concept word, rare disease supplementary concept word, unique identifier, synonyms]

29 or 30

exp Aldosterone/ or exp Renin-Angiotensin System/

exp Angiotensins/

(angiotensin II or angiotensin 2 or AII or Ang II or ATII or AT II or RAAS or renin angiotensin aldosterone system or aldosterone).mp. [mp=title, abstract, original title, name of substance word, subject heading word, floating sub-heading word, keyword heading word, organism supplementary concept word, protocol supplementary concept word, rare disease supplementary concept word, unique identifier, synonyms]

29 or 30

32 or 33 or 34

exp Oxidative Stress/ or exp Free Radicals/

exp Reactive Oxygen Species/

(reactive oxygen species or oxidative stress or free radical).mp. [mp=title, abstract, original title, name of substance word, subject heading word, floating sub-heading word, keyword heading word, organism supplementary concept word, protocol supplementary concept word, rare disease supplementary concept word, unique identifier, synonyms]

36 or 37 or 38

or/4-39

3 and 40
